# Determinants of violence towards care workers working in the home setting: A systematic review

**DOI:** 10.1002/ajim.23351

**Published:** 2022-03-29

**Authors:** Nang Nge Nge Phoo, Alison Reid

**Affiliations:** ^1^ School of Population Health, Faculty of Health Sciences Curtin University Bentley Western Australia Australia

**Keywords:** determinants, factors, home, home care, Type II, violence, workers

## Abstract

**Background:**

Home care is a rapidly growing industry. Violence towards home care workers is common, while also likely underreported. This violence adversely affects the physical and mental health of both workers and care recipients. The current study aims to identify and appraise recent evidence on the determinants of violence towards care workers working in the home setting.

**Methods:**

Six electronic databases: the Cumulative Index to Nursing and Allied Health Literature (CINAHL), EMBASE, Informit, Medline, PsycINFO, and Web of Science, were systematically searched. A systematic review was conducted in accordance with the Joanna Briggs Institute manual for evidence synthesis.

**Results:**

A total of 18 papers met the inclusion criteria. All were cross‐sectional surveys. The majority of studies were from the United States. The most commonly investigated associations were those between the medical history of clients, workers' apprehension of violence, worker‐client relationship, or care plans, and any form of violence or verbal abuse.

**Conclusion:**

Violence was common in clients with cognitive disorders, substance abuse disorder, and limited mobility; toward workers who feared that violence might happen; toward those who had very close or very distant worker‐client relationships; and when care plans were not inclusive of clients' needs. The current review highlights a gap in evidence on determinants of violence towards care workers working in the home setting, and suggests potential areas to be addressed to reduce such violence.

## 1 INTRODUCTION

Home care is a range of services provided by trained workers to people in their home setting to improve or maintain their quality of life.^1^ Home care recipients include, but are not limited to, the elderly, people with disabilities, and people with chronic illnesses.^1^ Globally, the home care market has been growing over the last few decades, and is projected to continue growing.^2^ In Australia, the number of home care recipients increased by 22% between March 2020 and March 2021.^3^ By March 2021, there were 167,124 active recipients of home care packages including assistance with daily living and health care in Australia.^3^ With a wide range of home care services such as housekeeping, personal care, social support, nursing care, and allied health services,^4^ the workers providing home care services varied widely.

Violence in the workplace was defined by the International Labor Organization (ILO) as “a range of unacceptable behaviors and practices, or threats thereof, whether a single occurrence or repeated, that aim at, result in, or are likely to result in physical, psychological, sexual or economic harm, and includes gender‐based violence and harassment.”^5^ The definition and scope of workplace violence differed across countries and based on research purposes.^5^, ^6^, ^7^, ^8^ A few examples are as follows:

- Verbal violence: yelling, using abusive words, making racial slurs or other types of discrimination, and verbal threats of harm, etc.
- Physical violence: pushing, scratching, pinching, slapping, punching, and throwing body fluids, etc.
- Sexual violence: unwanted sexual comments, unwanted body touch, and sexual assault, etc.
- Damage or loss of personal or work‐related materials: vandalism, robbery, or theft, etc.
- Income‐related violence: exploitation, fewer working hours, and loss of job, etc.
- Attacks by domestic animals.

Similarly, the classification of workplace violence differed across institutes or reports. The 2021 ILO report classified the violence as vertical—by employers, horizontal—by peer workers, and third parties—by clients.^5^ The 2001 workplace violence report by the Injury Prevention Research Center categorized the violence as Type I—with criminal intent, Type II—by clients, Type III—by fellow workers, and Type IV—by perpetrators who had personal relationship with workers.^8^

Violence towards care workers by clients or their families, Type II violence, is common in the home setting.^9^, ^10^, ^11^, ^12^ A meta‐analysis of 21 studies of home care workers from the United States (*n* = 12), Israel (*n* = 4), Japan (*n* = 2), Australia (*n* = 1), Canada (*n* = 1) and Ireland (*n* = 1) estimated the violence prevalence of 22.3% over the 12 months before the survey, and 30.2% over the carer's career.^9^ A review of 21 other studies, mostly from the United States (*n* = 8), looked at violence towards care workers both in home (*n* = 10) and institutional settings (*n* = 11). Between 33% and 87% of home care workers experienced verbal abuse from patients over the workers' career or while doing fieldwork.^10^ Both these reviews noted the diversity of the survey participants in terms of frequency of home visits, duration of each visit, nature of care, and interaction with care recipients.^9^, ^10^ Sexual abuse and sexual harassment was reported by 4% and 12%, respectively, from home care workers in a review of 14 studies of 6014 workers mostly from the United States (*n* = 5).^12^ Despite the high prevalence of violence against caregivers in the home setting, literature suggests that these incidents may be underreported.^13^, ^14^, ^15^ Reasons home care workers may not report violence include growing tolerance to violence, concerns of being blamed for the violence, losing working hours or their jobs, holding a temporary work visa, and unfamiliarity with legal system.^14^, ^16^, ^17^, ^18^, ^19^ Overall, it was not uncommon that home care workers were subject to different forms of violence perpetrated by clients.

Violent incidents have been shown to affect the workers' physical, mental and emotional health and to also impact the care recipients adversely.^20^, ^21^, ^22^, ^23^, ^24^ A survey of 1214 female home care workers in the United States reported a statistically significant association between exposure to any form of workplace violence and stress, depression, sleep disturbance and burnout.^20^ A review of nine studies that examined workers in home and institutional settings found a statistically significant association between physical violence and workers' mental health in nine out of 11 associations examined.^21^ Similarly, of the 13 associations examined between psychological violence and mental health, 11 statistically significant associations were reported.^21^ Furthermore, two studies included in the same review concluded that physical violence was significantly associated with musculoskeletal pain in workers.^21^ In addition to the direct impact of workplace violence on workers' health, there was an indirect impact on the care recipients. Among 823 home health aides in the United States, low job satisfaction and retention was associated with workplace violence.^22^ The poor health and high turnover rate of the workforce can adversely impact on the quality of care being provided.^23^, ^24^ Violence can lead to shortened or missed care visits and changes in care plans and this can have a negative impact on care outcomes.^23^, ^24^

Several determinants have been put forward by other authors as being associated with the poor safety of home care workers.^25^, ^26^ These include working alone in noninstitutional settings, inadequate health policies around home care, insufficient or a lack of record keeping and staff training, gaps between care planned for or received and the recipients' desire, and miscommunication among care recipients, providers and managers.^25^, ^26^ In addition, building designs not being conducive to care delivery, a lack of patient moving or handling equipment, and the location of client's homes in unsafe neighborhoods have also been suggested as factors associated with violence.^25^, ^26^ However, this is not a comprehensive list of possible determinants of violence as these studies have considered the safety of home care workers and not specifically determinants of the violence toward them.^25^, ^26^ In addition, there is limited experimental research around intervening factors that might reduce or prevent violence towards home care workers.^10^

Several literature reviews on the risk factors for workplace violence in institutional settings already exist. However, much less is known about those risk factors that are unique to carers working in their clients' homes.^27^, ^28^, ^29^, ^30^ In the home setting, clients and their families are in a position of power. Substance use cannot be banned, or weapons cannot be removed from the home setting. Care workers usually have to work alone in clients' homes with minimal or no support or protection by colleagues or managers. There is often a lack of appropriate equipment, for example, slide sheets, shower chairs, patient lifters and hoists, and this lack has been associated with patient violence. Moreover, domestic animals and unsafe neighborhood could impose threats to care workers. Given the numerous and varied range of adverse consequences following violence towards home care workers, and the unique risk factors of that violence, the aim of this study is to conduct a systematic literature review to identify determinants of violence that can be tested in an intervention study to reduce or prevent violence against home care workers at work.

## 2 MATERIALS AND METHODS

A systematic review was undertaken according to the Joanna Briggs Institute (JBI) Manual for Evidence Synthesis.^31^ Six electronic databases including Cumulative Index to Nursing and Allied Health Literature (CINAHL), EMBASE, Informit, Medline, PsycINFO, and Web of Science were searched systematically. The search was performed in August 2020 and updated in August 2021. The key words related to care workers working in the home setting and violence were used as search terms. The search terms were “home,” “home care,” “home healthcare,” “home health aide,” “direct care,” “personal care,” “nurse,” “nurs* assistant,” “social worker,” “violen*,” “workplace violence,” “client violence,” “safety,” “workplace safety,” “abuse,”, “harass*” “aggress*,” and “assault.”

The inclusion criteria were primary research studies conducted in high‐income countries, published in the peer‐reviewed literature, that examined factors associated with violence toward caregivers in the home setting, published in English during the years 2000 to July 2021. Only the studies from high‐income countries were included for the following reasons: having an established home care industry, an ageing population, a high number of women in workforce, and nuclear family structures in these countries.^32^ Home care workers were defined as those who

(1) assist care recipients with activities of daily living, e.g., personal care attendants, home care aides,
(2) provide health care, e.g., certified health care aides, home care nurses, or
(3) provide emotional support in the homes of clients, e.g., counselors, chaplains.

Home setting referred to the homes of clients. Violence towards care workers by clients or their friends or family members, that is, Type II violence, was included, regardless of the form of violence such as verbal, physical, sexual, property damage or loss, and exploitation, etc. Only quantitative studies which reported determinants of violence towards home care workers were included.

The exclusion criteria were care workers who did not provide hands‐on care to care recipients, family caregivers, or carers in aged care homes, nursing homes or long‐term care homes. Specifically excluded were studies that examined violence perpetrated on clients, violence caused by co‐workers, or that which took place during travel to clients' homes or in the neighborhood of clients' homes. Studies in low‐ and middle‐income countries and published before the year 2000 were also excluded, as were secondary analyses and reviews.

The search results were screened against inclusion and exclusion criteria by one of the two authors, and 10% of the search results were screened by the second author. The papers with relevant titles and abstracts were retrieved for full text check. A manual search of the bibliographies of relevant papers was undertaken to identify further studies not found in the literature search.

The methodological quality appraisal of the included studies was performed using the JBI critical appraisal instrument for systematic reviews of prevalence and incidence.^33^ The instrument assessed the presence of bias in study design, implementation, and data analysis. The instrument included nine criteria and each criterion was judged as “yes,” “no,” “unclear,” or “not applicable.” The quality assessment was performed by one of the two authors, and 10% of the included studies was appraised by the second author. Discordant appraisal comments were discussed, and agreement reached. When more than one paper reported the same study, the first published paper was appraised for methodological quality.

Data extraction was performed according to the JBI data extraction form for prevalence studies.^34^ The data extracted were the citation details including authors, title, journal, year, volume, and issue; generic study details such as study design, country, setting, timeframe for data collection, participant characteristics, violence types and prevalence, factors examined for associations with violence and descriptions of main results.

The determinants of violence towards the care workers working in the home setting were grouped into three groups: client factors, worker factors, and organizational factors. Due to the limited number of studies which examined associations between each factor and each type of violence, no meta‐analysis or sub‐group analysis was performed. The relevant findings of 18 included papers were critically appraised, narratively summarized, and a provisional conclusion was presented.

## 3 RESULTS

**Figure 1:**
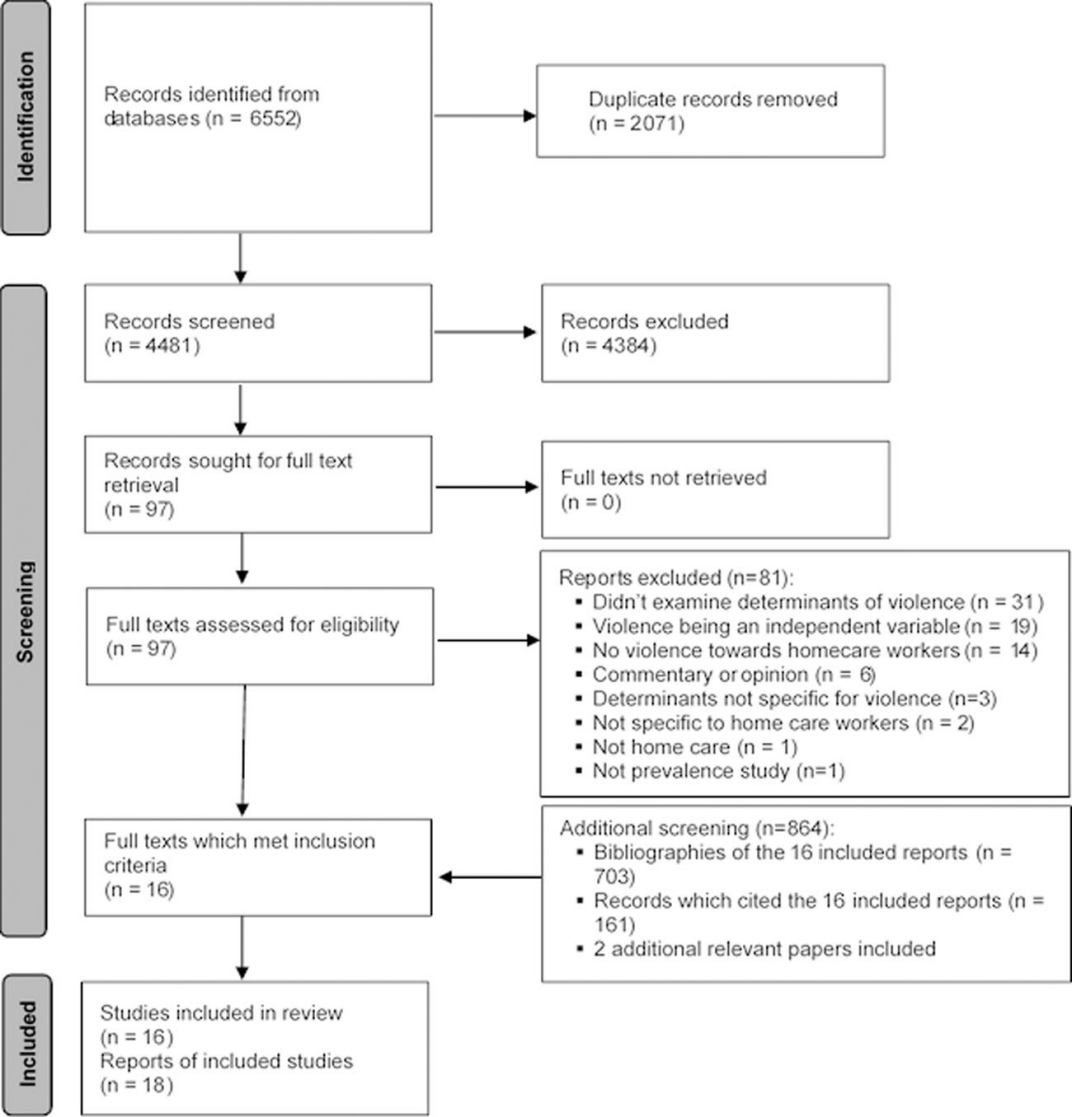
Search results of a systematic review of determinants of violence towards care workers working in the home setting, 2000–2021

A search of six databases resulted in 6552 manuscripts of which 2071 were duplicates. After screening the titles and abstracts of 4481 manuscripts, the full text of 97 papers were checked against the eligibility criteria. Initially, 16 papers met the inclusion criteria. A manual search of the bibliographies of those 16 papers did not yield any additional relevant papers. A manual search of the papers that cited those 16 papers resulted in two additional papers. Therefore, a total of 18 papers were included in the current review. Among the 18 papers included in the current review, five papers^6^, ^35^, ^36^, ^37^, ^38^ originated from two studies. As these papers presented different analysis results, all were included in the report about determinants, but only the first published papers of those studies were included in the narrative summary of general characteristics of studies and methodological quality appraisal. Therefore, a total of 18 papers from 15 studies were included in the review. The search and screening results are summarized in Figure 1.

### 3.1 Methodological quality of the studies reviewed

**Figure 2:**
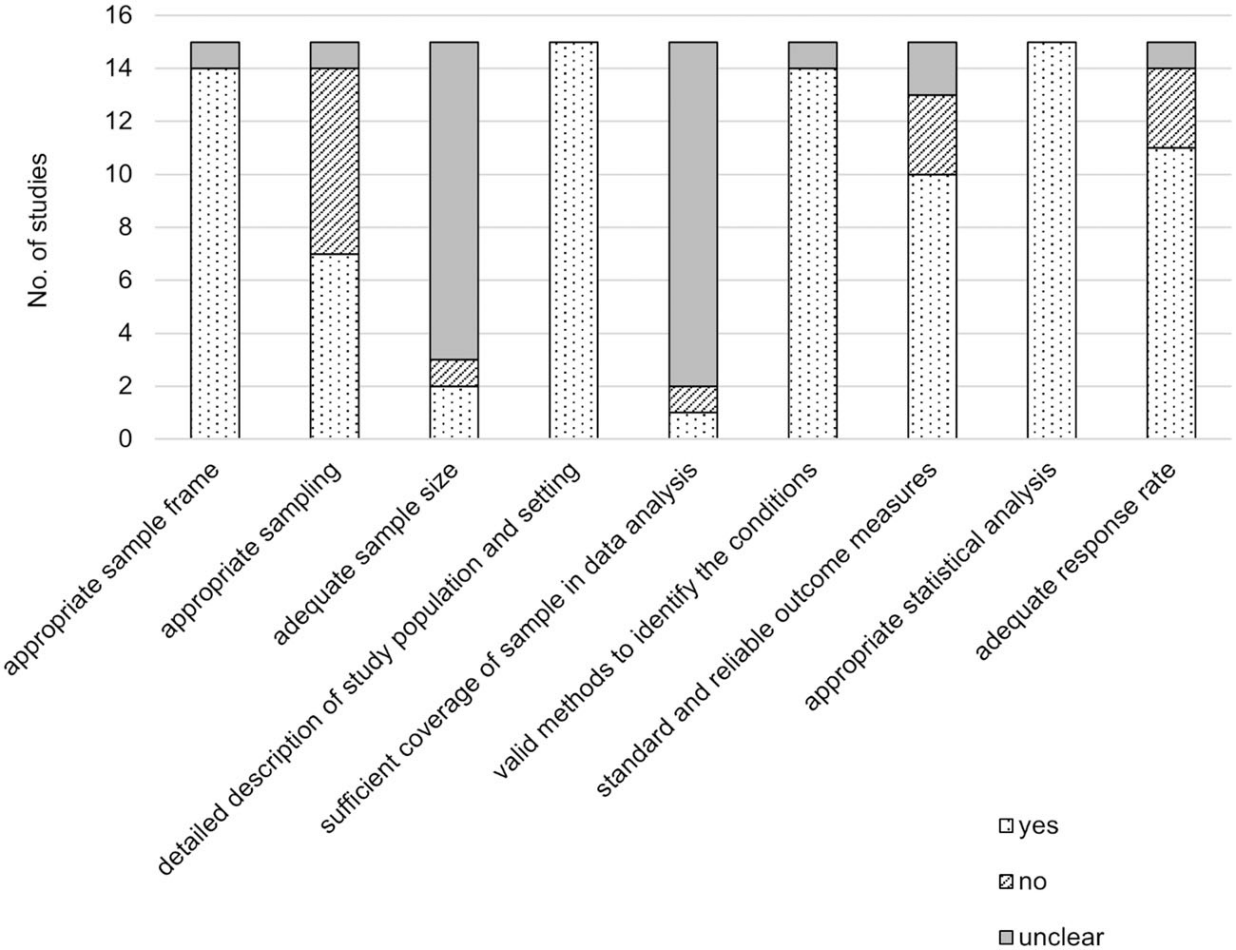
Methodological quality appraisal of the studies included in a systematic review of determinants of violence towards care workers working in the home setting, 2000–2021

Lists of the target population, for example, staff registers of home care agencies, were used as sampling frames in all (*n* = 14) but one study, in which recruitment was performed at the places frequented by Filipino migrants working in Israel^39^ (Figure 2). Convenience sampling from staff meetings and training sessions was performed in nearly half of the studies (*n* = 7); the remaining (*n* = 7) performed random sampling by inviting all care workers listed in the registers via mails or emails, or by performing systematic randomization; one study from Japan did not clearly report the sampling strategy.^40^ The required sample size was reported in only three studies,^11^, ^40^, ^41^ and the sample size reached was adequate in two of them.^11^, ^41^ All the studies described their study subjects and settings. The response rates for sub‐groups of participants were reported in only two studies,^16^, ^42^ and the rates were different across the groups with different socioeconomic status (SES) in the study from Australia, with 36% response rate in low SES capital city, 50% in high SES capital city and 54% in mixed‐SES noncapital city.^42^ Majority of the studies (*n* = 14) used valid methods to measure the conditions of interest. Magin et al.^42^ did not report details about the validity of the study questionnaire. The studies reported details about the validity of the questionnaire, and definitions of the variables used in the questionnaire. The majority of the studies (*n* = 10) applied the same measurement method for all the participants, that is, same recruitment approach, similar support to survey respondents regardless of survey modes, and trained interviewers. All the studies performed appropriate statistical analysis. The response rates were reported in the majority of the studies (*n* = 14), and it was less than 30% in three studies.^7^, ^40^, ^43^ Overall, nearly two‐thirds of the studies (*n* = 9) scored positively in six to eight out of nine quality appraisal criteria (Figure 2 and Table 1).

**Table 1:** Generic study characteristics of the papers included in a systematic review of determinants of violence towards care workers working in the home setting (2000–2021)

Reference	Country	Study design	Quality appraisal score (out of 9^a^)	*n*	Sample % of women	Age (years) (mean ± *SD*) (proportion)
Magin et al.^42^	Australia	Cross‐sectional	6	528	49.6%	51.3 ± 10.7
Galinsky et al.^28^	The United States	Cross‐sectional	5	677	92.0%	48 ± 12
McPhaul et al.^44^	The United States	Cross‐sectional	5	130	91.5%	53.8%: 50 years or older
Byon et al.^38^	The United States	Cross‐sectional	6	980	92.0%	45 ± 5.9
Quinn et al.^6^	The United States	Cross‐sectional	5	1249	87.0%	47^b^
Vladutiu et al.^7^	The United States	Cross‐sectional	4	191	Not reported	Not reported
Byon et al.^16^	The United States	Cross‐sectional	8	3377	95.0%	54.5%: 45 years or older
Byon et al.^35^	The United States	Cross‐sectional	–	964	92.0%	64%: 40 years and older
Green^39^	Israel	Cross‐sectional	4	187	86.8%	37.3 ± 6.4
Wong et al.^41^	Canada	Cross‐sectional	8	823	95.0%	48.6 ± 10.9
Fujimoto et al.^40^	Japan	Cross‐sectional	5	184	96.0%	45.4 ± 9.3
Green^45^	Israel	Cross‐sectional	6	523	86.9%	38.9 ± 8.5 (migrants)
						53.1 ± 10.6 (local)
Ifediora et al.^11^	Australia	Cross‐sectional	8	168	19.6%	41.1%: 39 years or less
Karlsson et al.^36^	The United States	Cross‐sectional	–	954	89.9%	48%: older than 48 years
Ridenour et al.^43^	The United States	Cross‐sectional	6	513	94.0%	81%: 40 years and older
Karlsson et al.^37^	The United States	Cross‐sectional	–	954	90.0%	48%: older than 48 years
Kim et al.^46^	Korea	Cross‐sectional	7	354	99.0%	50.1 ± 6.7
Mockli et al.^47^	Switzerland	Cross‐sectional	6	448	96.0%	44 ± 13.2

^a^ Each methodological quality criterion was appraised as one of the four categories: “yes,” “no,” “unclear,” or “not applicable.” Each “yes” score was counted as “1” and summed for reporting purpose.

^b^ Standard deviation not reported.

### 3.2 Characteristics of the studies reviewed

The study characteristics of the 18 papers reviewed from 15 studies are summarized in Table 1. All 15 studies were cross‐sectional surveys. Nearly half of the studies (*n* = 7) were conducted in the United States. There was a total of 10,332 participants in 15 studies, ranging from a minimum of 130 participants to a maximum of 3377. The response rate was reported in 14 studies, and it ranged from 17% to 84%.

### 3.3 Characteristics of the study participants

More than 80% of the study participants were females in the majority of the studies (*n* = 13). There were more male participants than females in the two studies which recruited medical doctors.^11^, ^42^ The mean age of the participants was around 45 years. The education level of the study participants was reported in eleven studies. More than half of the participants completed high school or less in five studies,^16^, ^35^, ^39^, ^43^, ^45^ and more than 70% of the participants attained college or higher education in another five studies.^11^, ^42^, ^44^, ^46^, ^47^ The remaining study reported that 64% of study participants held home care certificates.^6^ Among the five studies with a majority of participants completing high school or less, three reported the race of the participants, and the percentage of African American was considerably larger than that of Caucasian (80% vs. 17%, and 42% vs. 14%) in the two studies, respectively.^35^, ^43^ In contrast, in the study of which 88.5% of participants had a college degree or higher education, almost 80% were Caucasian.^44^ Race was reported in six of seven studies from the United States, and the proportion of African American ranged from 21% to 80%, Caucasians from 14% to 79.2%, and Asian 4% to 12%.^6^, ^16^, ^28^, ^37^, ^38^, ^43^ Among the eight papers from countries other than the United States, only one paper from Israel reported that all the study participants were from the Philippines.^39^

The job titles of study participants varied widely and included direct care workers in homes, home care aides, home care attendants, personal care attendants, personal care homemakers, companions, certified health care aides, nursing assistants, hospice aides, home care nurses, visiting nurse managers, home health and hospice care providers, general practitioners, physiotherapists, speech therapists, social workers, social work assistants, chaplains, and bereavement counselors. Collectively, 63.6% delivered assistance for daily living, 35.8% provided health care such as nursing care, treatment, speech therapy and physiotherapy, and 0.2% undertook social work. The mean duration of home care experience reported in five studies was 9.7 years.^38^, ^39^, ^40^, ^41^, ^46^ The mean working hours per week in three studies was 24.5 h per week.^6^, ^28^, ^39^ Among the five studies which reported the proportion of study participants who had full‐time or part‐time jobs, less than half of the participants had full‐time jobs in three studies.^11^, ^41^, ^47^

### 3.4 Factors associated with violence towards care workers working in the home setting

Various potential predictors were assessed for their association with violence towards care workers working in the home setting. The predictors examined by the reviewed studies were grouped into factors related to clients, workers, and organizations for reporting purposes (Tables 2, 3, 4). The outcome variables varied widely, for example, any form of violence, verbal abuse, physical assault, sexual harassment, emotional abuse, bullying, intimidation, exploitation, and injury due to violence (Tables 2, 3, 4). For reporting purposes, the outcome variables were grouped into “any form of violence” when they were not specified as verbal abuse, physical abuse, sexual harassment, emotional abuse, bullying, intimidation, exploitation, or injury. The variables grouped as ‘any form of violence' were most frequently tested since 13 papers reported 53 effect sizes. Verbal abuse (six papers and 26 effect sizes) was the second most common dependent variable in the reviewed studies. The effect sizes reported by the reviewed studies are summarized in Tables 2, 3, 4. The timeframe for experiencing violence was the past 12 months, with the current employer, or over their whole career. Overall, the heterogeneity between studies was large and only a few studies examined associations between similar independent and dependent factors.

**Table 2:** Client factors associated with violence towards care workers working in the home setting, a systematic review (2000–2021)

Author	Job titles	Predictors	Outcome variables	Statistics	Results
**1. Client factors**					
**1.1. Client demographic factors**					
Vladutiu et al.^7^	Aides, registered and licensed vocational nurses, therapists (occupational, physical, speech), social workers, Chaplains, or bereavement counselors, and other	Client's age	Violent events	Incidence rate per 1000 visit hours	<65 years old: 12.9 65–79 years old: 14.7 >80 years old: 22.8
Vladutiu et al.^7^		Client's sex	Violent events	Incidence rate per 1000 visit hours	26.2 (males) vs. 11.3 (females)
Vladutiu et al.^7^		Client's race	Violent events	Incidence rate per 1000 visit hours	18.9 (minority) vs. 16.2 (nonminority)
**1.2. Client illnesses and disorders**					
Galinsky et al.^28^	Home care aides, certified nursing assistants, and nurses	Any dementia patients	Assault prevalence in the past year	Percentage point difference	12% (with dementia) vs. 3% (no dementia)^a^
Byon et al.^38^	Direct care workers at home settings	Dual diagnosis with mental illness and substance abuse disorder	Act of physical violence	Crude OR (95% CI)	10.17 (3.91, 26.44)***
			Physical or verbal threat of violence	Crude OR (95% CI)	11.97 (6.17, 23.23)***
Vladutiu et al.^7^	Aides, registered and licensed vocational nurses, therapists (occupational, physical, speech), social workers, Chaplains or bereavement counselors, and others	Psychiatric disorder	Violent events	Incidence rate per 1000 visit hours	54.3 (with disorder) vs. 7.5 (no disorder)
		Substance abuse disorder	Violent events	Incidence rate per 1000 visit hours	32.8 (with disorder) vs. 14.3 (no disorder)
Green^39^	Migrant home care workers	Cognitive impairment	Work related abuse and exploitation	Incidence rate ratio: RR (95% CI)	2.45 (1.06, 5.64)**
		Cognitive impairment	Work related abuse and exploitation	Regression coefficient	0.89**
			No. of abuse incidents with current employer	Correlation coefficient	0.3**
Karlsson et al.^36^	Personal care attendant, personal care homemaker, homemaker, companion, home health aide, certified nursing assistant, and hospice aide	Dementia	Verbal abuse in the past 12 months	RR (95% CI)	1.65 (1.29, 2.10)^a^
		Mental illness or psychological disorders	Verbal abuse in the past 12 months	RR (95% CI)	1.58 (1.24, 2.01)^a^
		Limited mobility	Verbal abuse in the past 12 months	RR (95% CI)	1.73 (1.21, 2.47)^a^
**1.3. Client history of violence**					
Byon et al.^38^	Direct care workers at home settings	Client violent history	Act of physical violence	Crude OR (95% CI)	10.06 (4.12, 24.59)***
			Physical or verbal threat of violence	Crude OR (95% CI)	16.60 (9.20, 30.00)***
Vladutiu et al.^7^	Aides, registered and licensed vocational nurses, therapists (occupational, physical, speech), social workers, Chaplains or bereavement counselors, and others	History of violence	Violent events	Incidence rate per 1000 visit hours	145.4 (yes) vs. 10.2 (no)
Kim^46^	Visiting nurses	History of violence against visiting nurses	Workplace violence experience	OR (95% CI)	3.76 (1.97, 7.15)*
**1.4. Client home layout**					
Fujimoto et al.^40^	Registered nurses, and licensed practical nurses	Identifying the patient's home layout	Exposure to verbal abuse	Adjusted OR (95% CI)	2.6 (1.1, 6.1)*
				β	0.96
Karlsson et al.^36^	Personal care attendant, personal care homemaker, homemaker, companion, home health aide, certified nursing assistant, and hospice aide	Too little space made it hard to work	Verbal abuse in the past 12 months	RR	1.92 (1.51, 2.44)^a^
**1.5. Family and neighborhood**					
Vladutiu et al.^7^	Aides, registered and licensed vocational nurses, therapists (occupational, physical, speech), social workers, Chaplains or bereavement counselors, and others	Unsafe activity in the home or neighborhood	Violent events	Incidence rate per 1000 visit hours	38.0 (yes) vs. 14.3 (no)
Green^39^	Migrant home care workers	Live with clients only or also family members	Number of abuse incidents with current employer	Correlation coefficient	0.24**
**1.6. Geographical setting**					
Byon et al.^16^	Direct care workers in home settings	Micropolitan vs. Metropolitan (reference: Metropolitan)	Violence	OR (95% CI)	1.30 (0.42, 4.03)
Wong et al.^41^	Home care nurses	Geographical settings	Bullying/belittling/incivility	Percentage point difference	Rural: 8.1% Town: 3.3% Suburban: 3.2% Urban: 6.1%
			Potential for violence	Percentage point difference	Rural: 1.2% Town: 1.8% Suburban: 2.2% Urban: 4.0%
			Sexual harassment/abuse	Percentage point difference	Rural: 1.2% Town: 1.8% Suburban: 2.2% Urban: 4.0%

*Note*: *p < 0.05, **p < 0.01, ***p < 0.001.

Abbreviations: CI, confidence interval; OR, odds ratio; RR, risk ratio.

^a^ *p* value not reported in the original paper, hence statistical significance was observed based on CIs.

**Table 3:** Worker factors associated with violence towards care workers working in the home setting, a systematic review (2000–2021)

Author	Job titles	Predictors	Outcome variables	Statistics	Results
**2. Worker factors**					
**2.1. Worker socio‐demographic factors**					
Ifediora et al.^11^	Doctors (general practitioners, medical, surgical, emergency department, occupational physicians, pediatricians, public health)	Worker age (reference: ≥40 years old)	Any form of aggression in after‐hours house‐calls	OR (95% CI)	0.89 (0.36, 2.20)
Karlsson et al.^36^	Personal care attendant, personal care homemaker, homemaker, companion, home health aide, certified nursing assistant, and hospice aide	Worker age (reference: ≤48 years old)	Verbal abuse in the past 12 months	RR (95% CI)	0.59 (0.45, 0.76)^a^
Ridenour et al.^43^	Certified home health care aides	Worker age	Verbal assault	Proportion difference	20–29: 47*** 30–39: 35*** 40–49: 29*** 50–59: 22*** 60 and above: 18***
			Physical assault	Proportion difference	20–29: 33*** 30–39: 25*** 40–49: 20*** 50–59: 9*** 60 and above: 10***
			Sexual harassment	Proportion difference	20–29: 7* 30–39: 7* 40–49: 8* 50–59: 3* 60 and above – 1*
			Bullying/intimidation	Proportion difference	20–29: 17 30–39: 11 40–49: 8 50–59: 9 60 and above – 7
			Any assault	Proportion difference	20–29: 47 30–39: 42 40–49: 35 50–59: 32 60 and above: 26
Kim et al.^46^	Visiting nurses	Worker age	Workplace violence experience	OR (95% CI)	0.91 (0.86, 0.95)*
Byon et al.^38^	Direct care workers at home settings	Worker sex (reference: female)	Act of physical violence	Crude OR (95% CI)	3.25 (1.05, 10.10)*
		Worker sex (reference: female)	Physical or verbal threat of violence	Crude OR (95% CI)	1.76 (0.8, 3.88)
Byon et al.^16^	Direct care workers in home settings	Worker sex (reference: female)	Violence	OR (95% CI)	0.59 (0.06, 5.83)
Green^39^	Migrant home care workers	Worker sex (male vs. female)	Work‐related abuse and exploitation	Incidence RR (95% CI)	0.50 (0.26, 0.95)
			Work‐related abuse and exploitation	Regression coefficient	−0.70*
			No. of abuse with current employer	Correlation coefficient	−0.11
Ifediora.^11^	Doctors (general practitioners, medical, surgical, emergency department, occupational physicians, pediatricians, public health)	Worker sex (reference: female)	Any form of aggression in after‐hours house‐calls	OR (95% CI)	1.19 (0.39, 3.62)
Karlsson et al.^36^	Personal care attendant, personal care homemaker, homemaker, companion, home health aide, certified nursing assistant, and hospice aide	Worker sex (reference: female)	Verbal abuse in the past 12 months	RR (95% CI)	0.94 (0.62, 1.44)^a^
Ridenour et al.^43^	Certified home health care aides	Worker sex	Verbal assault	Proportion difference	Female: 26 Male: 30
			Physical assault	Proportion difference	Female: 15** Male: 36**
			Sexual harassment	Proportion difference	Female: 5 Male: 8
			Bullying/intimidation	Proportion difference	Female: 22 Male: 7
			Any assault	Proportion difference	Female: 33 Male: 44
Byon et al.^16^	Direct care workers in home settings	Worker education (reference: lower than high school)	Violence	OR (95% CI)	1.19 (0.37, 3.85)
Ifediora^11^	Doctors (general practitioners, medical, surgical, emergency department, occupational physicians, pediatricians, public health)	Worker education: postgraduate vocational status	Any form of aggression in after‐hours house‐calls	OR (95% CI)	0.28 (0.09, 0.84)*
Green^39^	Migrant home care workers	Worker financial situation	Work‐related abuse and exploitation	Incidence RR (95% CI)	0.55 (0.40, 0.75)***
			Work‐related abuse and exploitation	Regression coefficient	−0.61***
			No. of abuse with current employer	Correlation coefficient	−0.31**
Karlsson et al.^36^	Personal care attendant, personal care homemaker, homemaker, companion, home health aide, certified nursing assistant, and hospice aide	Economic dependence on job	Verbal abuse in the past 12 months	RR (95% CI)	1.18 (0.90, 1.55)^a^
Green^45^	Migrant home care workers	Years in Israel	Work‐related abuse and exploitation	Incidence RR (95% CI)	1.17 (1.06, 1.29)***
			Work‐related abuse and exploitation	Regression coefficient	−0.16***
			No. of abuse with current employer	Correlation coefficient	−0.16**
Karlsson et al.^36^	Personal care attendant, personal care homemaker, homemaker, companion, home health aide, certified nursing assistant, and hospice aide	Immigrant within past 5 years	Verbal abuse in the past 12 months	RR (95% CI)	1.01 (0.60, 1.69)^a^
Green^12^	Migrant live‐in care workers and local live‐out care workers	Migrant vs. local	Emotional abuse	Chi square	7.01**
				Percentage point difference	12.7% (migrants) vs. 5.4% (local)
			Physical abuse	Chi square	0.52
				Percentage point difference	1.2% (migrants) vs. 0.5% (local)
			Sexual abuse	Chi square	0.92
				Percentage point difference	1.2% (migrants) vs. 0.5% (local)
Karlsson et al.^36^	Personal care attendant, personal care homemaker, homemaker, companion, home health aide, certified nursing assistant, and hospice aide	Born outside the United States (reference: no)	Verbal abuse in the past 12 months	RR (95% CI)	yes: 0.91 (0.71, 1.17)^a^
Byon et al.^38^	Direct care workers at home settings	Race (reference: African American)	Act of physical violence	Crude OR (95% CI)	Non‐African American: 1.11 (0.36, 3.4)
			Physical or verbal threat of violence	Crude OR (95% CI)	Non‐African American: 0.74 (0.37, 1.50)
Byon et al.^16^	Direct care workers in home settings	Race (reference: Non‐Hispanic White)	Violence	OR (95% CI)	Non‐Hispanic Black: 0.32 (0.09, 1.16)
					Other: 0.46 (0.11, 1.82)
Karlsson et al.^36^	Personal care attendant, personal care homemaker, homemaker, companion, home health aide, certified nursing assistant, and hospice aide	Race (reference: White)	Verbal abuse in the past 12 months	RR (95% CI)	Non‐White or mixed race: 0.90 (0.69, 1.17)^a^
Ridenour et al.^43^	Certified home health care aides	Race	Verbal assault	Proportion difference	White: 31*** Black: 35*** Asian: 17*** Native American Indian: 50*** Hispanic/Latino: 15***
			Physical assault	Proportion difference	White: 17 Black: 22 Asian: 17 Native American Indian: 29 Hispanic/Latino: 9
			Sexual harassment	Proportion difference	White: 7 Black: 4 Asian: 4 Native American Indian: 29 Hispanic/Latino: 5
			Bullying/intimidation	Proportion difference	White: 18 Black: 7 Asian: 13 Native American Indian: 14 Hispanic/Latino: 7
			Any assault	Proportion difference	White 39 Black: 41 Asian: 24 Native American Indian: 50 Hispanic/Latino: 26
**2.2. Workers' tasks**					
Galinsky et al.^28^	Home care aides, certified nursing assistants, and nurses	Routine patient handling	Assault prevalence	Percentage point difference	7.77% (yes) vs. 0.66% (no)
Karlsson et al.^36^	Personal care attendant, personal care homemaker, homemaker, companion, home health aide, certified nursing assistant, and hospice aide	Use any client handling/transfer device	Verbal abuse in the past 12 months	RR (95% CI)	1.36 (1.06, 1.75)^a^
**2.3. Work experience**					
Green^39^	Migrant home care workers	Years of home care experience	Work‐related abuse and exploitation	Incidence rate ratio (95% CI)	0.97 (0.83, 1.12)
				Regression coefficient	−0.03
			Number of abuse incidents with current employer	Correlation coefficient	−0.22**
**2.4. Apprehension of violence**					
Magin, et al.^42^	General practitioners	Apprehension about violence during business hours (often)	Experience of violence in the past 12 months	Percentage point difference	None: 0.0%** Low: 72.7%** High: 27.3%**
		Apprehension about violence during business hours (sometimes)	Experience of violence in the past 12 months	Percentage point difference	None: 23.9%** Low: 57.8%** High: 18.3%**
		Apprehension about violence during business hours (almost never)	Experience of violence in the past 12 months	Percentage point difference	None: 39.0%** Low: 48.6%** High: 12.4%**
		Apprehension about violence during after business hours (often)	Experience of violence in the past 12 months	Percentage point difference	None: 16.2%*** Low: 59.5%*** High: 24.3%***
		Apprehension about violence after business hours (sometimes)	Experience of violence in the past 12 months	Percentage point difference	None: 24.5%*** Low: 60.2%*** High: 15.3%***
		Apprehension about violence after business hours (almost never)	Experience of violence in the past 12 months	Percentage point difference	None: 37.8%*** Low: 50.4%*** High: 11.8%***
Galinsky et al.^28^	Home care aides, certified nursing assistants, and nurses	Felt threatened by others	Assault prevalence	Percentage point difference	14.49% (yes) vs. 3.52% (no)^a^
Ifediora^11^	Doctors (general practitioners, medical, surgical, emergency department, occupational physicians, pediatricians, public health)	Apprehension over aggression	Any form of aggression in after‐hours house‐calls	OR (95% CI)	3.99 (1.54, 10.31)**
**2.5. Worker—client relationship**					
Vladutiu, et al.^7^	Aides, registered and licensed vocational nurses, therapists (occupational, physical, speech), social workers, Chaplains, or bereavement counselors, and other	First visit to the home vs. Not the first visit	Violent events	Incidence rate per 1000 visit hours	9.0 (first visit) vs. 21.8 (not the first visit)
Byon et al.^16^	Direct care workers in home settings	Language barrier	Injury due to patient violence	OR (95% CI)	4.44 (1.57, 12.56)**
Byon et al.^35^	Direct care workers in home settings	Overly involved class vs. nonfamilial class	Physical assaults	Probability	Overly involved class = 0.11 (0.06, 0.18) vs. nonfamilial class = 0.04 (0.02, 0.07)
Green^39^	Migrant home care workers	Years with current employer	Work‐related abuse and exploitation	Incidence rate ratio (95% CI)	0.79 (0.67, 0.93)***
				Regression coefficient	−0.24***
			No. of abuse with current employer	Correlation coefficient	−0.25**
Karlsson et al.^36^	Personal care attendant, personal care homemaker, homemaker, companion, home health aide, certified nursing assistant, and hospice aide	Language discordance (reference: no)	Verbal abuse in the past 12 months	RR (95% CI)	1.16 (0.87, 1.55)^a^
Kim et al.^46^	visiting nurses	Temporary employment vs. permanent employment	Workplace violence experience	OR (95% CI)	2.95 (1.10, 7.88)*
**2.6. Workers' personal affairs**					
Mockli et al.^47^	Home care workers, certified assistant nurse, certified nurse aides, registered nurse, and licensed practical nurses	Work stressors	Aggression	Correlation coefficient	0.29***
		Work‐family conflicts	Aggression	Correlation coefficient	0.21***

*Note*: *p < 0.05, **p < 0.01, ***p < 0.001.

Abbreviations: CI, confidence interval; OR, odds ratio; RR, risk ratio.

^a^ *p* value not reported in the original paper, hence statistical significance was observed based on CIs.

**Table 4:** Organizational factors associated with violence towards care workers working in the home setting, a systematic review (2000–2021)

Author	Job titles	Predictors	Outcome variables	Statistics	Results
**3. Organizational factors**					
**3.1. Care plans**					
Fujimoto et al.^40^	Registered nurses and licensed practical nurses	Care adjustments to decrease the risk in case of patients posing a violence risk	Any form of abuse	Adjusted OR (95% CI)	2.4 (1.1, 5.2)*
				β	0.90
			Sexual harassment	Adjusted OR (95% CI)	4.0 (1.1, 14.7)*
				β	1.61
Karlsson et al.^36^	Personal care attendant, personal care homemaker, homemaker, companion, home health aide, certified nursing assistant, and hospice aide	Did not have the time I needed	Verbal abuse in the past 12 months	RR (95% CI)	1.60 (1.17, 2.18)^a^
		Did not have a clear care plan	Verbal abuse in the past 12 months	RR (95% CI)	1.62 (1.21, 2.17)^a^
		Predictable working hours vs. nonpredictable	Verbal abuse in the past 12 months	RR (95% CI)	0.65 (0.51, 0.83)^a^
Karlsson et al.^36^	Personal care attendant, personal care homemaker, homemaker, companion, home health aide, certified nursing assistant, and hospice aide	Being asked to do things not part of the job	Verbal abuse	Prevalence ratio (95% CI)	1.93 (1.47, 2.52)^a^
			Physical/sexual abuse	Prevalence ratio (95% CI)	1.81 (1.13, 2.91)^a^
**3.2. Type of employers**					
Fujimoto^11^	Registered nurses and licensed practical nurses	Care adjustments to decrease the risk in case of patients posing a violence risk	Any form of abuse	Adjusted OR (95% CI)	2.4 (1.1, 5.2)*
Quinn et al.^6^	Personal care attendant, personal care homemaker, homemaker, companion, home health aide, certified nursing assistant, and hospice aide	Agency‐hired vs. Client‐hired	Any physical violence	Percentage point difference	7.9% (agency‐hired) Vs 5.2% (client‐hired)
			Any verbal violence	Percentage point difference	23.2% (agency‐hired) Vs 14.3% (client‐hired)^***^
Byon et al.^16^	Direct care workers in home settings	Agency type: for profit vs. other (reference: for profit)	Violence	OR (95% CI)	1.25 (0.32, 4.86)
Karlsson et al.^36^	Personal care attendant, personal care homemaker, homemaker, companion, home health aide, certified nursing assistant, and hospice aide	Hire type: client vs. agency (reference: agency)	Verbal abuse in the past 12 months	RR (95% CI)	0.76 (0.58, 1.00)^a^
Ridenour et al.^43^	Certified home health care aides	Types of employers	Verbal assault	Proportion	Assisted living: 49^***^ Home health agency: 18^***^ Hospice: 31^***^ Personal care home: 43^***^
			Physical assault	Proportion	Assisted living: 36^***^ Home health agency: 10^***^ Hospice: 20^***^ Personal care home: 22^***^
			Sexual harassment	Proportion	Assisted living: 7 Home health agency: 3 Hospice: 10 Personal care home: 4
			Bullying/intimidation	Proportion	Assisted living: 15^***^ Home health agency: 5^***^ Hospice: 18^***^ Personal care home: 19^***^
			Any assault	Proportion	Assisted living: 53^***^ Home health agency: 26^***^ Hospice: 45^***^ Personal care home: 0^***^
**3.3. Training**					
Byon et al.^16^	Direct care workers in home settings	Training on abusive patient (reference: no/poor/fair training)	Violence	OR (95% CI)	0.55 (0.18, 1.69)
		Training on dementia patient (reference: no/poor/fair training)	Violence	OR (95% CI)	3.47 (0.98, 12.27)
Fujimoto et al.^40^	Registered nurses and licensed practical nurses	Providing education about violence and training for its prevention	Verbal abuse	Adjusted OR (95% CI)	4.9 (1.2, 20.2)*
				β	1.94
**3.4. Measures related to violence**					
Fujimoto et al.^40^	Registered nurses and licensed practical nurses	Management of visiting schedules and confirmation of the home visiting nurses' locations during visits	Any form of violence	Adjusted OR (95% CI)	0.4 (0.2, 0.9)*
				β	−0.95

*Note*: *p < 0.05, **p < 0.01, ***p < 0.001.

Abbreviations: CI, confidence interval; OR, odds ratio; RR, risk ratio.

^a^ *p* value not reported in the original paper, hence statistical significance was observed based on CIs.

### 3.5 Client factors

The most commonly assessed client factor was the medical history of clients (Table 2). Associations between client's illnesses or disorders and any form of violent events were examined in four studies,^7^, ^28^, ^38^, ^39^ and five effect sizes were reported. Associations between illnesses and verbal abuse was examined in one study,^36^ and three effect sizes were reported. The illnesses or disorders included dementia, mental illness, cognitive impairment, psychiatric disorder, substance abuse disorder, and limited mobility. Byon et al.'s^38^ paper of 876 direct care workers in the home setting in Chicago, the United States reported that the workers caring for clients with a dual diagnosis of mental illness and substance abuse disorder had a 12 times higher chance of being assaulted than those caring for clients without such disorders (odds ratio [OR]: 11.97, 95% confidence interval [CI]: 6.17, 23.23, *p* < 0.001). Likewise, Green et al.'s^39^ study of 187 Filipino home care workers in Israel reported a weak but significant correlation (correlation coefficient 0.3, *p* < 0.01) between cognitive impairment of clients and number of abuse incidents. The risk of both professional and paraprofessional workers being abused was higher when clients had dementia or cognitive impairment in the studies by Galinsky et al.^28^ and Vladutiu et al.^7^ from the United States which reported percentage point differences. Similarly, in the Karlsson et al.'s^36^ paper, the risk of home care aides getting verbally abused was higher when the clients had dementia, mental illnesses, psychological disorders, or limited mobility.

Other client factors assessed in the studies included demographic factors, history of violence, home layout, family and neighborhood, and geographical setting (Table 2). Only the study by Vladutiu et al. examined the association between clients' age, sex and race and violence.^7^ The violence rate was higher among clients of older age, male sex and minority race.^7^ Clients who had perpetrated violence towards care workers in the past were more likely to do the same again in the studies by Byon et al.,^38^ Vladutiu et al.,^7^ and Kim et al.^46^ Verbal abuse was common among nurses who identified the layout of the client's home before the first visit in the study by Fujimoto et al.^40^ Violence was more common when there was limited space to work in the clients' homes in Karlsson et al.'s^36^ paper. The workers in Vladutiu et al.'s^7^ study experienced more violence when the clients' neighborhood was unsafe. Green et al.^39^ reported that more violence took place when migrant home care workers lived with the clients and their family members than with the clients only.

### 3.6 Worker factors

The most tested worker factors were worker sex, workers' fear about violence, and worker‐client relationship. Of the five associations examined between worker sex and any form of violence, none were statistically significant.^11^, ^35^, ^38^, ^39^, ^43^ Male workers in the Byon et al.'s^38^ study and Ridenour et al.'s^43^ study were more prone to physical abuse than females in both effect sizes reported. The workers' apprehension about violence was examined for its associations with any form of violence in three studies^11^, ^28^, ^42^ (Table 3). Magin et al.^42^ examined associations between general practitioners who never or sometimes or often worried about violence during or after business hours and no or low or high level of experience of violence. Low level of violence, that is, verbal abuse, property damage or theft, threats or slander, was most common with any category of fear, and the associations were statistically significant.^42^ Similarly, Galinsky et al.'s^28^ and Ifediora's^11^ studies, one each from the United States and Australia, reported that there was a higher risk of experiencing violence when workers worried that violence would happen. The association between close worker‐client relationship and violent events was direct in the studies by Vladutiu et al.^7^ and Byon et al.,^35^ and inverse in the studies by Green et al.^39^ and Kim et al.^46^ There was a higher chance of care workers being abused when they became more familiar with their clients in the Vladutiu et al.'s^7^ and Byon et al.'s^35^ studies from the United States. Conversely, migrant home care workers in the Green et al.'s^39^ and home visiting nurses in Kim et al.'s^46^ studies from Israel and Korea reported higher experience of violence in their early years with clients or for temporary employment. The chance of getting injured due to patient violence was high when there were language barriers between direct care workers and clients in Byon et al.'s^16^ study.

Other worker factors examined in the included studies were socio‐demographic factors, job tasks, work experience and personal affairs (Table 3). Violence was generally more common among young workers in three studies, as verbal abuse was more common among home care aides aged less than 48 years compared with those aged 48 years and older in the Karlsson et al.'s^36^ paper from the United States. Similarly, verbal and physical violence were most common among certified home health care aides aged 20–29 years in the Ridenour et al.'s^43^ study from the United States. There was a decreasing chance of violence with increasing age among home visiting nurses in the Kim et al.'s^46^ study from Korea. In Ifediora's^11^ study, people tended to display aggressive behaviors towards medical doctors without postgraduate vocational status, for example, fellowship of the Royal Australian College of General Practitioners, than doctors with such vocational status. In the study by Green et al.,^39^ migrant care workers experienced more violence in their early years in the receiving country, and when they had financial difficulty. When compared to the local care workers, migrant care workers in Israel were more susceptible to violence than local workers in the Green et al.'s^45^ study. Regarding the worker's race, there was a statistically significant association with verbal violence in the study by Ridenour et al., native American Indian (proportion of experiencing verbal assault = 50) had a higher risk of incurring verbal violence at work than certified home health care aides who self‐identified as Black, White, Asian and Hispanic or Latino (proportion = 35 and lower).^43^ The association between race and violence was not significant in the studies by Byon et al.^16^,^38^ and Karlsson et al.^36^ When the workers' task involved handling or transfer of clients, they experienced more assaults in the Galinsky et al.'s^28^ and Karlsson et al.'s^36^ studies. In Mockli et al.'s^47^ study, care workers who had work stress and work‐family conflicts were prone to face aggression in the workplace.

### 3.7 Organizational factors

Only six papers examined organizational factors as determinants of violence. These items included care plans, the type of agency or employer, whether workers had undertaken violence management training and other measures taken by nursing agencies to address violence (Table 4). The associations between the practicality of the care plan and violence was examined in a survey among home care aides who provided assistance for daily living in the United States, and reported in two separate papers by Karlsson et al.^36^, ^37^ There was a higher risk of aides being verbally abused when the care plan was not clear, did not allocate adequate time, required working at nonpredictable hours, or when the aides were asked by clients to do tasks which were not their role.^36^, ^37^ Regarding the types of employer, agency‐hired workers reported more violence than client‐hired workers in Quinn et al.'s^6^ study. On comparing the workplace in Ridenour et al.'s^43^ study, certified home health aides working for assisted living homes incurred more verbal, physical and any assault than those undertaking personal home care, hospice care, or those who worked for a home health agency. Workers undertaking personal home care had a higher risk of being bullied or intimidated than in any other workplace.^43^ In the study by Fujimoto et al.,^40^ the odds of experiencing verbal abuse was 4.9 times more common (adjusted OR: 4.9, 95% CI: 1.2, 20.2, *p* < 0.01) among nurses who worked for the agencies which provided violence prevention training. When nurses had to visit psychiatric patients in their homes, violence preventive measures such as adjusting care plans (adjusted OR: 2.4, 95% CI: 1.1, 5.2, *p* < 0.05) and confirmation of nurses' location during home visits (adjusted OR: 0.4, 95% CI: 0.2, 0.9, *p* < 0.05) were significantly associated with exposure to any form of violence, while provision of violence management training was associated with increased risk of incurring verbal abuse in Fujimoto et al.'s^40^ study.

## 4 DISCUSSION

Across all 15 studies examined in this review, violence towards home care workers was a commonly reported phenomena. The determinant most commonly associated with violence perpetrated against these workers was the physical and mental health of their clients, e.g., cognitive impairment, substance abuse disorder, or limited mobility,^7^, ^28^, ^36^, ^38^, ^39^ wherein home carers of clients with those attributes had a greater risk of incurring violence. This finding concurs with the results of other systematic reviews that found aggression was common among clients with Alzheimer disease, dementia, and mild cognitive impairment.^48^, ^49^ Despite the frequency of violence reported, there was a gap in training and support for home care workers caring for clients with dementia.^50^

Another determinant of violence towards home care workers was their fear of violence.^11^, ^28^, ^42^ Magin et al.^42^ suggested that the fear alone could not be the causal factor for experiencing aggression and suggested that after‐hours visits to homes could be the cause. Galinsky et al.^28^ made a similar suggestion that the relation between fear and violence could be due to objective factors, e.g., unsafe neighborhood. Ifediora^11^ commented that it was natural that those who had experienced violence would be concerned that it could happen again. Such worry led to general practitioners undertaking fewer or no after‐hours home visits in Magin et al.'s^42^ study from Australia.^42^ Furthermore, almost the same percentage of workers who felt the threat of violence (20.6%) shortened the length of their home visits as did those who had actually been assaulted (22.6%).^28^

Too much or too little familiarity with clients was also a determinant of violence towards the home care workers. Violence could occur both in the early stages of a worker‐client relationship as well as in longer term relationships. The higher abuse rates incurred during the early stages of the worker‐client relationship might have been because of nonfamiliarity between workers and clients, and the workers' perception that each violent event was serious.^39^ In contrast, Vladutiu et al.^7^ suggested that worker‐client familiarity which exceeded the professional relationship might lead to the workers becoming more tolerant of violence and the clients assuming a normalization of violence, thus leading to more violent events over time. Moreover, poor worker‐client relationship could adversely impact on the quality of care received by clients.^51^, ^52^

Another determinant of violence against home care workers was when the care plan did not meet clients' expectations, or when the time allocated for care was inadequate. Unclear care plans could lead to misunderstandings between workers and clients and result in violence.^36^ Moreover, there could be violence even with clear care plans when such plans did not meet the clients' needs, and when workers or clients did not adhere to the plans.^30^ A review of 15 observational studies about adult home care quality commented that time constraints in care plans could hinder clients from getting what they want.^52^ The same review concluded that workers spending more time than the time allocated in the care plans might be associated with good quality care.^52^

There are limitations in the current review. All the reviewed studies were from high‐income countries. Most of the reviewed studies were conducted in the United States, and only one or two studies in other countries. Hence, it is not possible to generalize the review findings to a wider population in different countries, or particularly in lower income countries. The search was limited to peer‐reviewed journal articles published in English. Therefore, relevant papers published in languages other than English, dissertations, theses, and grey literature might have been missed. Despite the small number of studies which met the eligibility criteria, the predictors and outcome variables were very diverse, as were the statistics used to examine the associations. This hindered performing a meta‐analysis or sub‐group analysis, and also affected the rigor of provisional conclusions. As the predictors and outcome variables were subjectively measured in the reviewed studies, under‐ or over‐estimates might have affected the reports around associations.

Some of the determinants of violence against home care workers identified in the current review could be trialed in future interventions to reduce such violence. For example, for care workers delivering care to clients with cognitive disorder, substance use disorder and limited mobility, tools for how to work safely with such clients could be integrated into compulsory workplace health and safety training. For care workers who have too close or too distant a relationship with clients, compulsory workplace health and safety training could include information on how to maintain professional relationship boundaries with clients. Those care workers who fear violence should be encouraged to seek help from the home care agencies. In addition, there have to be systems in place to ensure these agencies adopt accountable violence management procedures. For the care workers who have to endure the consequences of the gaps between the care plans offered by the agencies and the care demanded by clients, the agencies need to continually monitor that the clients' expectation of care match the reality provided by the care workers.

The determinants of violence towards care workers working in the home setting include certain medical conditions of the clients, fear of violence by workers, a too familiar or new worker‐client relationship, and inadequate or not adhered to care plans. This provisional conclusion has to be viewed by taking into account the small number of studies examining each association. The current review highlighted a paucity of studies examining factors associated with violence against care workers working in the home setting, and in particular too much heterogeneity in the outcomes that were measured, or the covariates examined. But as a priority, the determinants of violence identified in this review should be tested as to their effectiveness at reducing violence against home care workers in intervention programs, to reduce or prevent this common occupational health and safety hazard.

## CONFLICTS OF INTEREST

The authors declare no conflicts of interest.

## DISCLOSURE BY AJIM EDITOR OF RECORD

John Meyer declares that he has no conflict of interest in the review and publication decision regarding this article.

## AUTHOR CONTRIBUTIONS

**Alison Reid**: identified the gaps in literature relating to the topic of the current study. Under her supervision, **Nang Nge Nge Phoo**: conducted a systematic review and drafted the manuscript. Reid cross‐checked each phase of the review process, and finalized the manuscript.

## Data Availability

All the papers reviewed in this study are available.
